# Shedding new light on the Crab with polarized X-rays

**DOI:** 10.1038/s41598-017-07390-7

**Published:** 2017-08-10

**Authors:** M. Chauvin, H.-G. Florén, M. Friis, M. Jackson, T. Kamae, J. Kataoka, T. Kawano, M. Kiss, V. Mikhalev, T. Mizuno, N. Ohashi, T. Stana, H. Tajima, H. Takahashi, N. Uchida, M. Pearce

**Affiliations:** 1 KTH Royal Institute of Technology, Department of Physics, 106 91 Stockholm, Sweden; 2 The Oskar Klein Centre for Cosmoparticle Physics, AlbaNova University Centre, 106 91 Stockholm, Sweden; 3 Stockholm University, Department of Astronomy, 106 91 Stockholm, Sweden; 4 University of Tokyo, Department of Physics, Tokyo, 113-0033 Japan; 5 SLAC/KIPAC, Stanford University, 2575 Sand Hill Road, Menlo Park, CA 94025 USA; 6 Research Institute for Science and Engineering, Waseda University, Tokyo, 169-8555 Japan; 7 Hiroshima University, Department of Physical Science, Hiroshima, 739-8526 Japan; 8 Institute for Space-Earth Environment Research, Nagoya University, Aichi, 464-8601 Japan; 9 Present Address: School of Physics and Astronomy, Cardiff University, Cardiff, CF24 3AA UK

## Abstract

Strong magnetic fields, synchrotron emission, and Compton scattering are omnipresent in compact celestial X-ray sources. Emissions in the X-ray energy band are consequently expected to be linearly polarized. X-ray polarimetry provides a unique diagnostic to study the location and fundamental mechanisms behind emission processes. The polarization of emissions from a bright celestial X-ray source, the Crab, is reported here for the first time in the hard X-ray band (~20–160 keV). The Crab is a complex system consisting of a central pulsar, a diffuse pulsar wind nebula, as well as structures in the inner nebula including a jet and torus. Measurements are made by a purpose-built and calibrated polarimeter, PoGO+. The polarization vector is found to be aligned with the spin axis of the pulsar for a polarization fraction, PF = (20.9 ± 5.0)%. This is higher than that of the optical diffuse nebula, implying a more compact emission site, though not as compact as, e.g., the synchrotron knot. Contrary to measurements at higher energies, no significant temporal evolution of phase-integrated polarisation parameters is observed. The polarization parameters for the pulsar itself are measured for the first time in the X-ray energy band and are consistent with observations at optical wavelengths.

## Introduction

**Figure 1:**
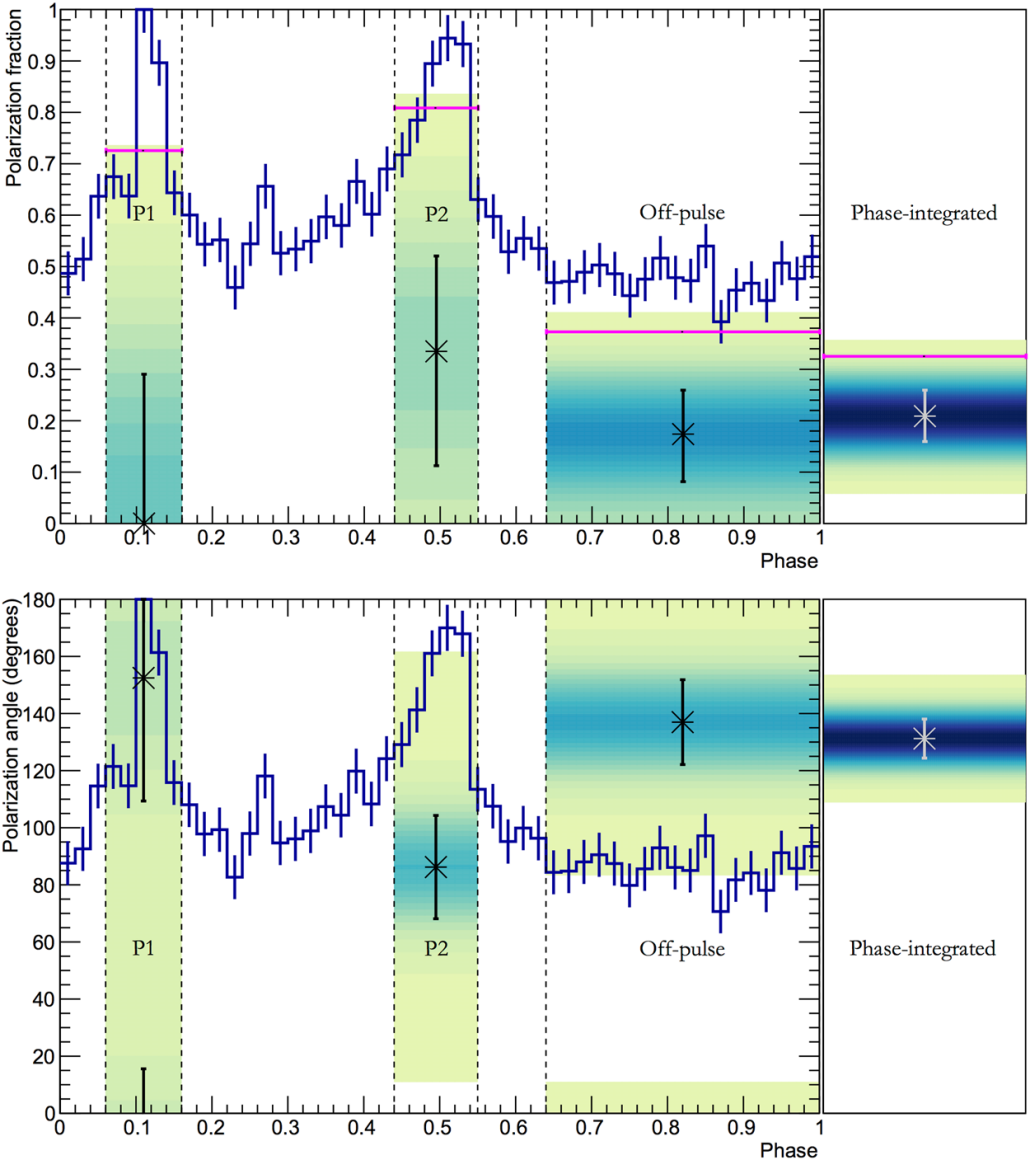
Light-curve and polarization results for the Crab. Results for the polarization fraction (top) and polarization angle (bottom) are super-imposed on the observed light-curve. The right-most column shows phase-integrated results. Colored overlays show the probability density distribution ﻿(see Supplementary Information) for the corresponding part of the light-curve (P1, P2, off-pulse, respectively). The off-pulse has been subtracted from P1 and P2 yielding a pure pulsar contribution. The error bars show the marginalized one standard-deviation Gaussian probability content while the magenta lines correspond to 99% upper limits (applicable to the polarization fraction only). The highest number of light-curve counts (corresponding to the peak of P1) is 2519.

The Crab is a prototypical celestial particle accelerator^1^. The central pulsar comprises a highly magnetized (~10^8^ T) neutron star. The rotation period, 33.7 ms, slows as Ṗ = 4.2 × 10^−13^ ^2^. Of order 1% of the rotational energy loss is imparted to electrons (and positrons, here referred to as electrons) which can be accelerated up to an energy of several PeV (10^15^ eV). Electrons are extracted along the boundary of the co-rotating magnetosphere and directed along open magnetic field lines to the light cylinder. The electrons pass through turbulent magnetic fields near and beyond the light cylinder and form an ultra-relativistic wind. As this wind expands into ejecta from the progenitor star and supernova explosion, wind termination shocks are formed. Resulting synchrotron and inverse Compton interactions generate the high luminosity (~1.3 × 10^38^ erg/s) arcminute-sized nebula^1^. The pulsar wind nebula shows a wealth of smaller-scale structures which are known to be highly dynamic, varying on short time-scales, and in emission energy. Overall, the size of the emitting region decreases when observed at high energies, but X-ray emission is still observed even close to the boundary of the nebula^3^, evidence that the situation is complex. The observations presented here cover a large field-of-view, ~2°, encompassing the pulsar and entire nebula. The rotational phase of the pulsar is used to isolate the pulsar emissions from that of the nebula. The phase-folded light-curve as observed by PoGO+^4^ in the ~20–160 keV range is shown in Fig. 1. It is obtained by folding through a pulsar rotation period using the closest ephemeris to our observations from the Jodrell Bank Observatory^5^. The peaks arise due to the offset between the rotation and magnetic axes of the pulsar. Two peaks are apparent - a main peak, P1, phase interval 0.06–0.14, and a second peak P2, phase interval 0.44–0.55. The phase intervals are defined in the same way as in ref. 6. The pulsar light-curve is reproduced by a variety of models, e.g. refs 7 and 10, where X-rays are generated in synchrotron emission from accelerated electrons. The polarization properties of the pulsar emission depend on the location of the emission region in the magnetosphere^11^. Emissions dominated by the nebula can be isolated by selecting X-rays from the off-pulse region, phase interval 0.64–1.0.

The optical polarization properties for a handful of rotation-powered pulsars have been determined. For the Crab, averaged over all phases, PF = (9.8 ± 0.1)% for the pulsar region^12^. Phase-resolved polarimetry reveals rapid swings in the polarization angle, PA, and significantly reduced PF in both pulse peaks. This supports caustic emission from the outer magnetosphere^9^, where the peak emission comprises radiation from a large range of altitudes (i.e. emission with different field directions, hence the swing in PA and destructive interference of PF), and/or emission from the equatorial current sheet where the magnetic field changes polarity^10^. X-ray polarization is expected to track that observed at optical wavelengths since both emissions are synchrotron in nature, share the same magnetic field-lines and the same electron population^11^. High-energy pulsar models are currently tested using optical polarization data. Until now, X-ray results stem solely from a polarimeter on-board OSO-8^13^. Highly statistically significant results have been obtained for the nebula. A PF of (19.2 ± 1.0)% at a PA of (156.4 ± 1.4)° and (19.5 ± 2.8)% at a PA of (152.6 ± 4.0)° was determined at 2.6 and 5.2 keV, respectively. There is only marginal evidence of polarization for the pulsed part of the light curve^14^. At higher energies (>200 keV), polarimetric measurements have been reported by INTEGRAL, as described in the discussion below. The CZTI instrument on-board AstroSat^15^ is expected to provide polarimetric data (>100 keV) there-by also complementing the results presented here.

## Results

**Figure 2:**
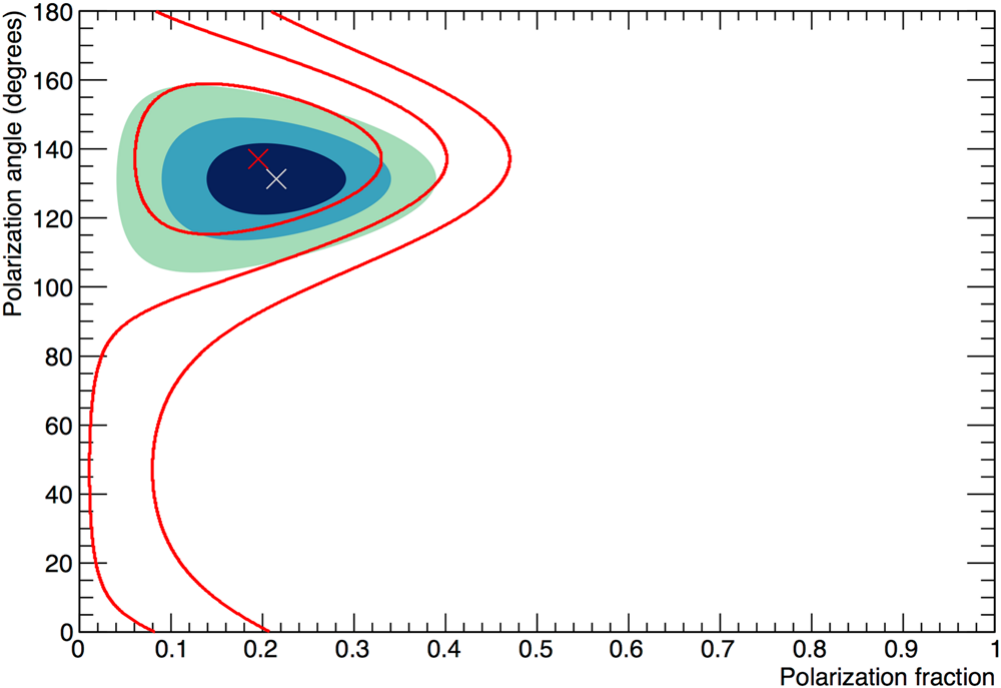
Polarisation contour plots for the Crab observation. Gaussian 1, 2 and 3𝜎 probability contours for phase-integrated (shaded area) and off- pulse Crab observations (red lines). Crosses indicate maximum a posteriori estimates, see Supplementary Information for details.

Results are presented in Figs 1 and 2. Phase-integrated Crab emissions exhibit PF = (20.9 ± 5.0)%, providing a detection at more than 4σ significance. For synchrotron processes, the maximum allowed PF for a uniform magnetic field geometry is 60–75%^16^, for electron spectral indices in the range 1–3^6^. Despite observations encompassing both the pulsar and the topologically complex wind nebula, a relatively high value of PF is found, indicating a magnetically ordered, and therefore compact, emission site. High-resolution X-ray images from Chandra^17^ reveal a rich structure in the inner nebula. Two concentric magnetic tori are centered on the pulsar position. The inner torus lies in a plane perpendicular to the pulsar spin axis, whose projection onto the sky is (124.0 ± 0.1)° ^18^. All angles are defined anticlockwise relative to North (i.e. to the East). Electrons trapped in the toroidal magnetic field produce synchrotron radiation with a PA parallel to the pulsar spin axis^19^. A PA value of (131.3 ± 6.8)° is determined, which coincides with that of the spin axis. This is in agreement with the expectation from NuSTAR imaging^20^ showing the toroidal ring region dominating emission in the hard X-ray band of PoGO+. Optical polarization measurements have higher spatial resolution, which allows individual features to be discerned. Measurements with HST^21^ find a high polarization fraction from the synchrotron knot, PF = (59.0 ± 1.9)%, at a PA = (124.7 ± 1.0)°, as well as in the wisps at PAs of 124–130°. In contrast, a vector map of the entire inner nebula shows a peak distribution of PA around 165°. X-ray imaging cannot resolve such details, but the coincidence of the PA with observed structures points to these features being associated with the X-ray torus.

For PoGO+, the light-curve pulsed fraction contributes (18.5 ± 0.5)% to the total observed flux. Phase-integrated measurements are therefore indeed nebula-dominated, as conjectured above. The off-pulse region exhibits PF = (${17.4}_{-9.3}^{+8.6}$)% and PA = (137 ± 15)°. The polarization properties of this nebula-dominated phase are compatible with the phase-integrated properties. Emissions from the pulsar peaks are isolated through phase selections and the constant off-pulse contribution from the nebula is subtracted. For P1, the 99% upper limit for PF is 70%. The PA is poorly constrained, see the Supplementary Information. For P2, PF = (${33.5}_{-22.3}^{+18.6}$)% and PA = (86 ± 18)°.

## Discussion

The SPI and IBIS instruments on board INTEGRAL have been used as polarimeters. It is important to note that neither instrument rotated during observations (see Methods, below), and rely on Monte Carlo simulations to resolve angular dependencies in the instrument. This makes the determined polarization parameters vulnerable to systematic errors. Moreover, pre-launch polarimetric calibration has not been performed. The SPI team reports PF = (46 ± 10)% for PA = (123 ± 11)° for the off-pulse period of the Crab pulsar in the energy band 100 keV–1 MeV^22^. The IBIS team reports a phase-integrated result of PF = (${47}_{-13}^{+19}$)% for PA = (100 ± 11)° for 200–800 keV^23^. More recent IBIS measurements reported a change in PA after a Crab flare event which may indicate the presence of magnetic field reconnection^24^. However, the measured PF is surprisingly large at >60%.

**Figure 3:**
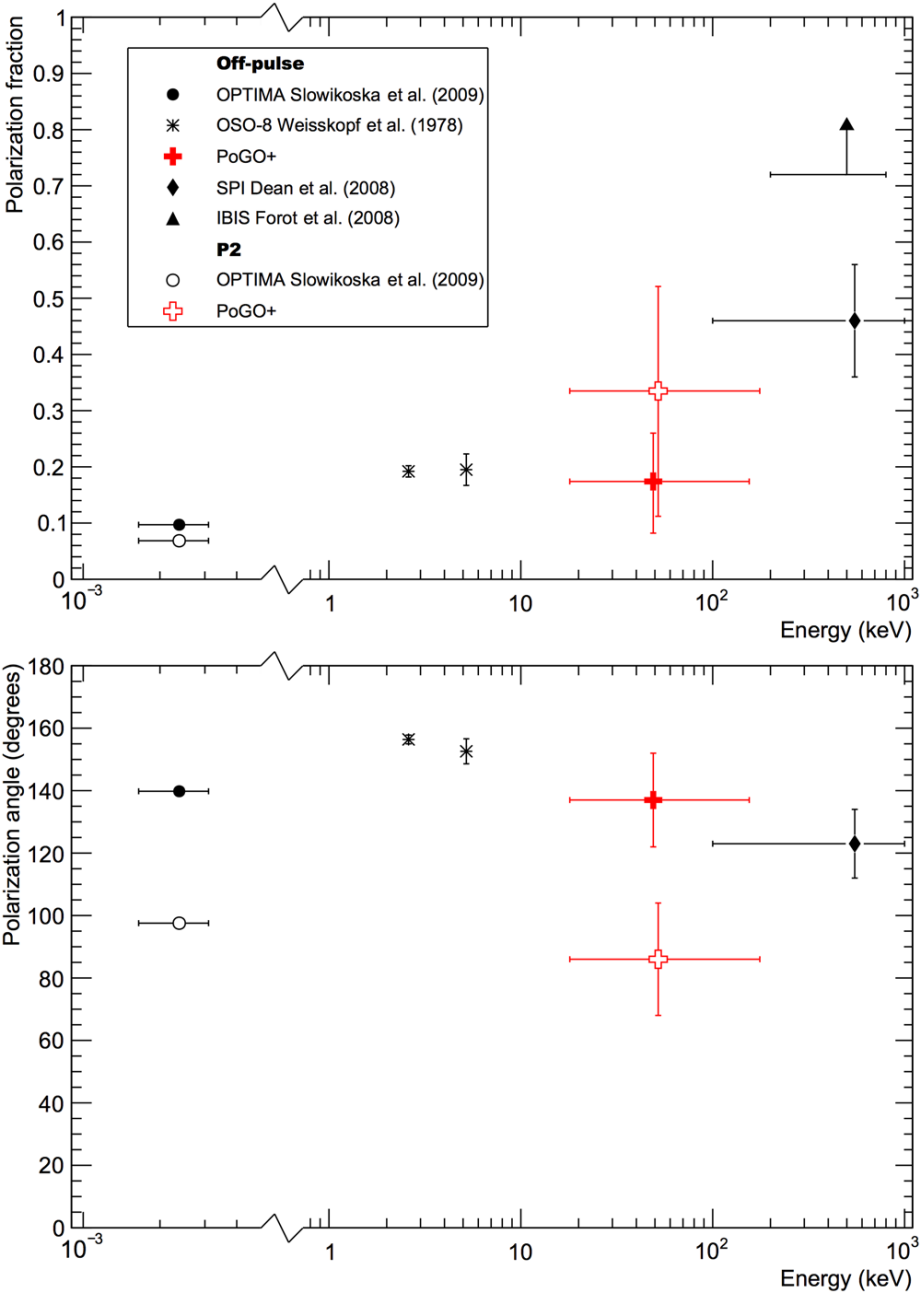
Comparison to other polarimetric studies of the Crab nebula (off-pulse) and P2. Data is shown for the PF (top row) and the PA (bottom row). It is noted that for optical results the nebula is spatially separated whereas temporal separation is applied in the X-ray regime.

Figure 3 summarizes existing off-pulse polarimetric observations of the Crab at high energies. Our off-pulse results show no significant changes in PF compared to that observed at optical wavelengths for the nebula. Considering the inconsistency of results between SPI and IBIS for similar energy ranges, our results favour the slower increase in PF with energy reported by SPI.

The P2 (off-pulse subtracted) measurements are the first in the hard X-ray regime. It is encouraging that similar behavior to optical observations is seen, as this regime is used to validate high-energy models, e.g. ref. 25 found that only the polarization for the bridge emission between P1 and P2 was expected to change significantly with energy. In X-rays, the polarization parameters are integrated over the entire pulse which complicates interpretation. For comparison, measurements of P2^12^ with OPTIMA at the Nordic Optical Telescope, when integrated over the corresponding phase-region, yield PF = (6.85 ± 0.2)% and PA = (97.6 ± 0.2)°. The superior temporal resolution shows an angle swing through the peak of 100° (130° for P1).

**Figure 4:**
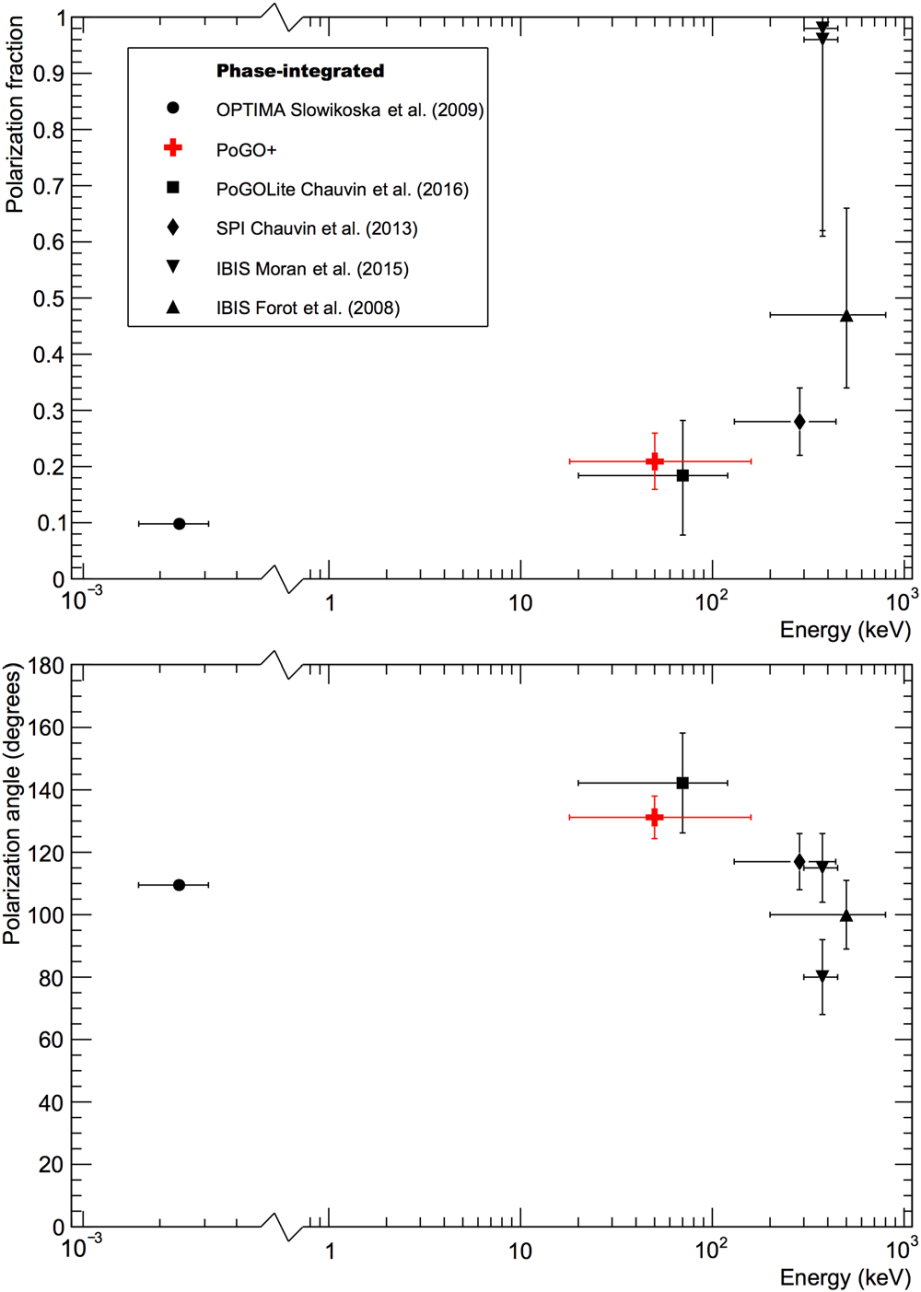
Comparison to other phase-integrated polarimetric studies of the Crab.

Our off-pulse PA is also consistent with that reported in the optical regime and is parallel to the pulsar spin axis^18^ as expected. This contrasts with the PA measured by OSO-8 which is 30–33° displaced from the spin axis. INTEGRAL instruments also reported off-pulse PAs consistent with the spin axis. Newer measurements^24^ claim a >3σ difference with a phase-integrated PA of (80 ± 12)°. Off-pulse measurements have not been reported. Figure 4 also includes measurements from the PoGOLite Pathfinder instrument, a predecessor to PoGO+^26^, performed in 2013. The measurements are 3 years apart (as compared to ~8 for IBIS). Although the detection of polarization by PoGOLite is marginal, no significant change is observed in either PF or PA for this energy interval.

## Summary

A significant detection of polarized emission from the Crab system (including phase dependence) in the energy interval ~20–160 keV is reported for the first time. Measurements do not support the high PF value^24^ derived using an X-ray instrument onboard INTEGRAL as a polarimeter. Moreover, no significant change in polarization angle is observed when comparing PoGO+ data to that obtained 3 years previously by PoGOLite. The PA for the Crab nebula is observed to be consistent with the projection of the pulsar spin axis in the plane of the sky, compatible with an origin in the toroidal magnetic field. It is tentatively confirmed that optical polarization data near the pulsar can be used as a proxy for higher energy emission when constructing pulsar models.

## Methods

PoGO+ observations were conducted in July 2016 from a stabilized balloon-borne platform in the upper stratosphere (~40 km altitude)^27^. The polarimeter detection volume comprises an array of 61 plastic scintillator rods, each with hexagonal cross-section (12 cm long, ~3 cm wide). The 2° field-of-view of each rod is defined by a collimator. Polarized X-rays will Compton scatter preferentially in the direction perpendicular to the electric field vector^28^. A polarization event is defined by exactly 2 interactions in the scintillator array. Each event defines an azimuthal scattering angle in the plane of the sky. The distribution of angles is a harmonic function, the phase of which defines PA. The modulation amplitude defines PF. For an unpolarized beam, PF is Rayleigh-distributed and therefore positive definite. As a result, a large number of photons is required to make a statistically constrained measurement. Additionally, the design of the polarimeter must include a method to distinguish instrumental effects from source polarization. For PoGO+, this is achieved by rotating the polarimeter around the viewing axis during observations. This generates a continuous distribution of scattering angles and provides a uniform polarimetric response. The symmetric detector geometry and rotation allows the scattering angle distribution to be determined without the need for corrections from computer models.

The field-of-view is centered on the Crab with a precision better than 0.05° during observations. The effective area for polarization measurements is 3.8 cm^2^ at 50 keV. Performance characteristics of the polarimter are detailed in the Supplementary Information. The polarization sensitivity (‘modulation factor’) for a 100% polarized beam is (37.8 ± 0.7)%^4^. Due to the positive definite nature of measurements, it is particularly important that the polarimeter response is determined using X-ray beams of known polarization, as well as unpolarized beams. The modulation factor for an unpolarized radiation source with an energy comparable to the median energy registered during Crab observations is (0.10 ± 0.12)%. Background impinging from outside the collimated field-of-view is mitigated with a segmented anticoincidence system and a passive polyethylene neutron shield. A residual background arises predominantly from neutrons scattered into the detection volume from the atmosphere^29^.

A total of 594419 polarization events were identified during 92 ks of Crab observations. The signal-to-background ratio is 0.142. Anisotropic background may cause a fake polarization signal. To address this, interspersed observations, totaling 79 ks, are conducted of fields 5° to the East and West of the Crab. Transition between Crab and background fields occurs every ~15 minutes, in order to track temporal behaviour. Polarization parameters are derived using unbinned and background-subtracted Stokes parameters^30^, as described in the Supplementary Information.

## Electronic supplementary material

Supplementary Information
