# Publisher Correction: Shedding new light on the Crab with polarized X-rays

**DOI:** 10.1038/s41598-018-24853-7

**Published:** 2018-05-17

**Authors:** M. Chauvin, H.-G. Florén, M. Friis, M. Jackson, T. Kamae, J. Kataoka, T. Kawano, M. Kiss, V. Mikhalev, T. Mizuno, N. Ohashi, T. Stana, H. Tajima, H. Takahashi, N. Uchida, M. Pearce

**Affiliations:** 1 KTH Royal Institute of Technology, Department of Physics, 106 91 Stockholm, Sweden; 2 The Oskar Klein Centre for Cosmoparticle Physics, AlbaNova University Centre, 106 91 Stockholm, Sweden; 3 Stockholm University, Department of Astronomy, 106 91 Stockholm, Sweden; 4 University of Tokyo, Department of Physics, Tokyo, 113-0033 Japan; 5 SLAC/KIPAC, Stanford University, 2575 Sand Hill Road, Menlo Park, CA 94025 USA; 6 Research Institute for Science and Engineering, Waseda University, Tokyo, 169-8555 Japan; 7 Hiroshima University, Department of Physical Science, Hiroshima, 739-8526 Japan; 8 Institute for Space-Earth Environment Research, Nagoya University, Aichi, 464-8601 Japan; 9 Present Address: School of Physics and Astronomy, Cardiff University, Cardiff, CF24 3AA UK

Correction to: *Scientific Reports* 10.1038/s41598-017-07390-7, published online 10 August 2017

This Article contains a typographical error in the legend of Figure 2.

“Gaussian 1, 2 and 3𝜎”

should read:

“Gaussian 1, 2 and 3σ”

